# A pleural drainage tube misplaced into the right atrium

**DOI:** 10.1186/s13054-023-04343-7

**Published:** 2023-02-04

**Authors:** Yiping Feng, Shouyin Jiang, Shanxiang Xu, Xiao Lu

**Affiliations:** 1 Department of Emergency Medicine, Second Affiliated Hospital, Zhejiang University School of Medicine, Hangzhou, 310009 China; 2 Key Laboratory of The Diagnosis and Treatment of Severe Trauma and Burn of Zhejiang Province, Hangzhou, 310009 China; 3 Zhejiang Province Clinical Research Center for Emergency and Critical Care Medicine, Hangzhou, 310009 China; 4 Research Institute of Emergency Medicine, Zhejiang University, Hangzhou, 310009 China

**Dear Editor**,

Common complications of thoracentesis included pain, bleeding, pneumothorax and infection [1]. Cardiac perforation has been reported as a possible complication of thoracentesis in patients with cardiomegaly or distortion of normal human anatomy [2].

A 70-year-old female presented to our department due to requiring for extracting a pleural tube penetrated into the right heart 22 days ago. She was previously hospitalized for the right femur fracture. The patient had rheumatic heart disease with mitral and tricuspid valves replaced. She suffered deoxygenation following internal femur fixation. Bedside ultrasound operator regarded improperly the enlarged right atrium as pleural effusion. A pleural tube was placed but drained massive dark red fluid shortly. Chest computerized tomography (CT) confirmed misplaced tube into the right atrium. Repeat chest CT in our hospital revealed that a large mural thrombus developed in the right atrial wall with the catheter tip stuck in (Fig. 1A and B). A right-sided thoracotomy with careful tube extraction followed by purse-string suture was performed 2 days after admission (Fig. 1C). No active hemorrhage or pulmonary embolism was observed after surgery (Fig. 1D).

**Fig. 1:**
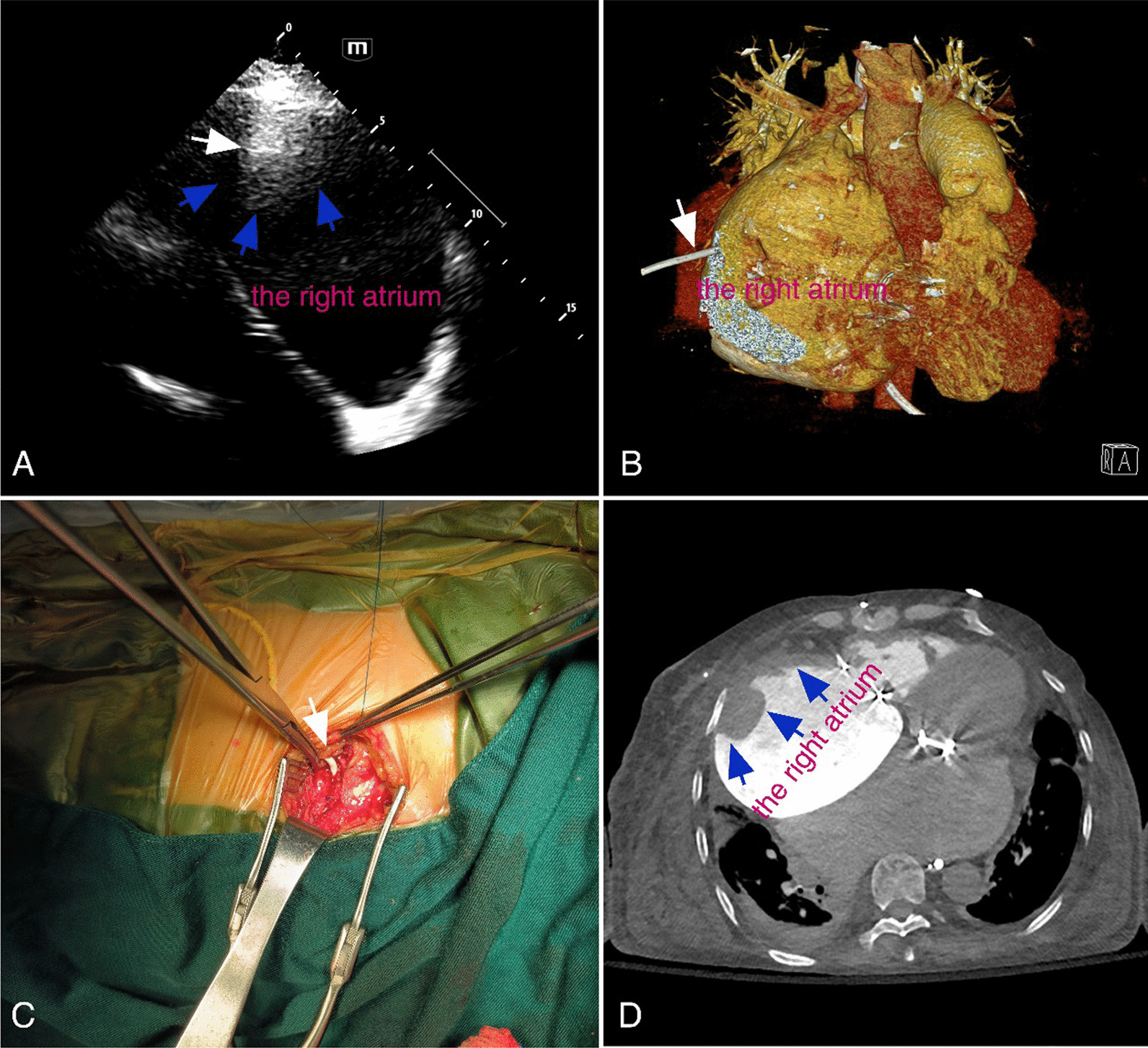
**A** Transthoracic echocardiogram demonstrating thrombus (blue arrows) in the right atrial wall with high-echo catheter tip stuck in (white arrow); **B** 3D CT reconstruction showing outer segment of the tube; **C** intraoperative finding of drainage tube inserted into the heart; **D** residual flake-like mural thrombus (blue arrows) in the right atrial wall after tube extraction

Misplacement of the right pleural tube into the heart is extremely rare, which raises caution that a thorough thoracic pre-procedural evaluation cannot be overlooked [3]. The definitive therapy for extraction of iatrogenic and sterile cardiac foreign body can be managed alternatively and safely with delayed surgery. Post-surgical anticoagulation is essential for prophylaxis of pulmonary embolism.

## Data Availability

The datasets used and/or analyzed during the current study are available from the corresponding author on reasonable request.
